# Progress in protecting vestibular hair cells

**DOI:** 10.1007/s00204-021-03067-3

**Published:** 2021-05-13

**Authors:** Luoying Jiang, Zhiwei Zheng, Yingzi He

**Affiliations:** 1 ENT Institute and Department of Otorhinolaryngology, Eye and ENT Hospital, State Key Laboratory of Medical Neurobiology and MOE Frontiers Center for Brain Science, Fudan University, Shanghai, 200031 China; 2 NHC Key Laboratory of Hearing Medicine, Fudan University, Shanghai, 200031 China

**Keywords:** Vestibular dysfunction, Hair cells, Ototoxic drugs, Protection

## Abstract

Vestibular hair cells are mechanosensory receptors that are capable of detecting changes in head position and thereby allow animals to maintain their posture and coordinate their movement. Vestibular hair cells are susceptible to ototoxic drugs, aging, and genetic factors that can lead to permanent vestibular dysfunction. Vestibular dysfunction mainly results from the injury of hair cells, which are located in the vestibular sensory epithelium. This review summarizes the mechanisms of different factors causing vestibular hair cell damage and therapeutic strategies to protect vestibular hair cells.

## Introduction

Peripheral vestibular organs are composed of two otolith organs: a utricle and a saccule, and three semicircular canals with different orientations: anterior, posterior, and horizontal semicircular canals, where vestibular hair cells (HCs) are mechanosensory receptors that are capable of detecting the position changes of the head so that animals can adjust their posture and coordinate their movement (Merchant et al. 2000). Healthy HCs are therefore essential for performing vestibular physiological functions. Among the population, vestibular dysfunction is prevalent, and it increases remarkably with age (Agrawal et al. 2009). In the US, about 18% of the population aged 40–49 years, and 49% of the population aged 60–69 years suffer from vestibular dysfunction. The morbidity of this disorder arrives at 80% among people over 80 years old in the US (Negishi-Oshino et al. 2019).

Vestibular HCs are vulnerable to gene factors, aging, and exposure to therapeutic drugs, such as aminoglycoside and cisplatin. Extensive loss of vestibular sensory cells can cause dysfunction of the peripheral vestibular apparatus, eliciting multiple unpleasant symptoms, such as imbalance, instability, dizziness, and vertigo (Burns and Stone 2017). With symptoms getting more severe, patients are likely to become depressed due to a general decline in their physical functions (Brosel and Strupp 2019). These symptoms may exert a negative impact on one’s quality of life and increase the health care expenditure of a society.

However, research on the inner ear is mainly focused on the cochlea, while the damage factors and protective strategies of vestibular HCs have received relatively little attention. Furthermore, there are currently no equivalent therapeutic strategies for vestibular dysfunction compared to some effective hearing loss treatments such as cochlear implants and hearing aid devices (Wall et al. 2002). Therefore, how to protect vestibular HCs from loss and injury is an important issue.

## Structural and functional features of vestibular hair cells

**Fig. 1:**
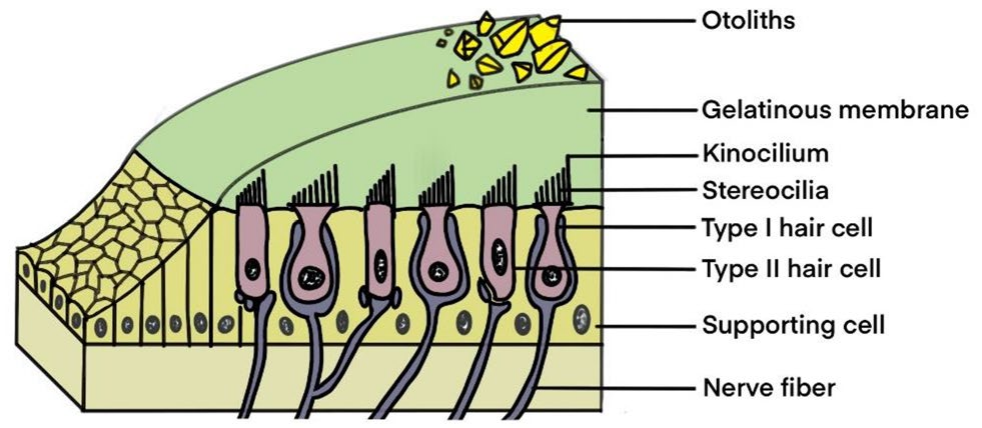
Structure of the macula. The macula is composed of the otolithic membrane and macular epithelium. The otolithic membrane consists of a gelatinous membrane and some otoliths. The macular epithelium comprises sensory hair cells and supporting cells

**Fig. 2:**
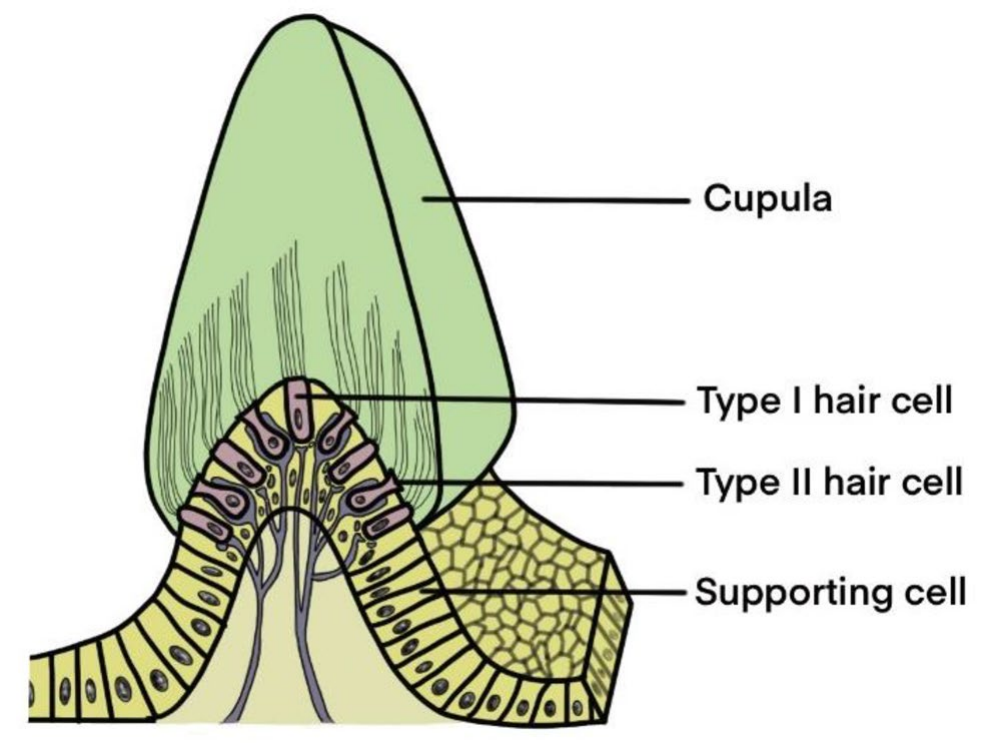
Structure of the crista ampullaris. The histological morphology of the crista ampullaris is similar to the macula, while its gelatinous membrane called the cupula has no otoliths and is thicker than that of the macula

The sensory epithelium of the otolith organ, called macula, detects linear or translational acceleration, and the sensory epithelium of the semicircular canals, called the crista ampullaris, senses angular acceleration (Kingma and van de Berg 2016). The macula is composed of the otolithic membrane and the macular epithelium (Jeong et al. 2020). The otolithic membrane consists of a gelatinous membrane and some otoliths consisting of calcium carbonate (Kingma and van de Berg 2016) and the macular epithelium comprises sensory HCs and supporting cells (Jeong et al. 2020) (Fig. 1). The histological morphology of the crista ampullaris is similar to the macula, while its gelatinous membrane called cupula has no otoliths, and is thicker than that of the macula (Kingma and van de Berg 2016) (Fig. 2).

**Fig. 3:**
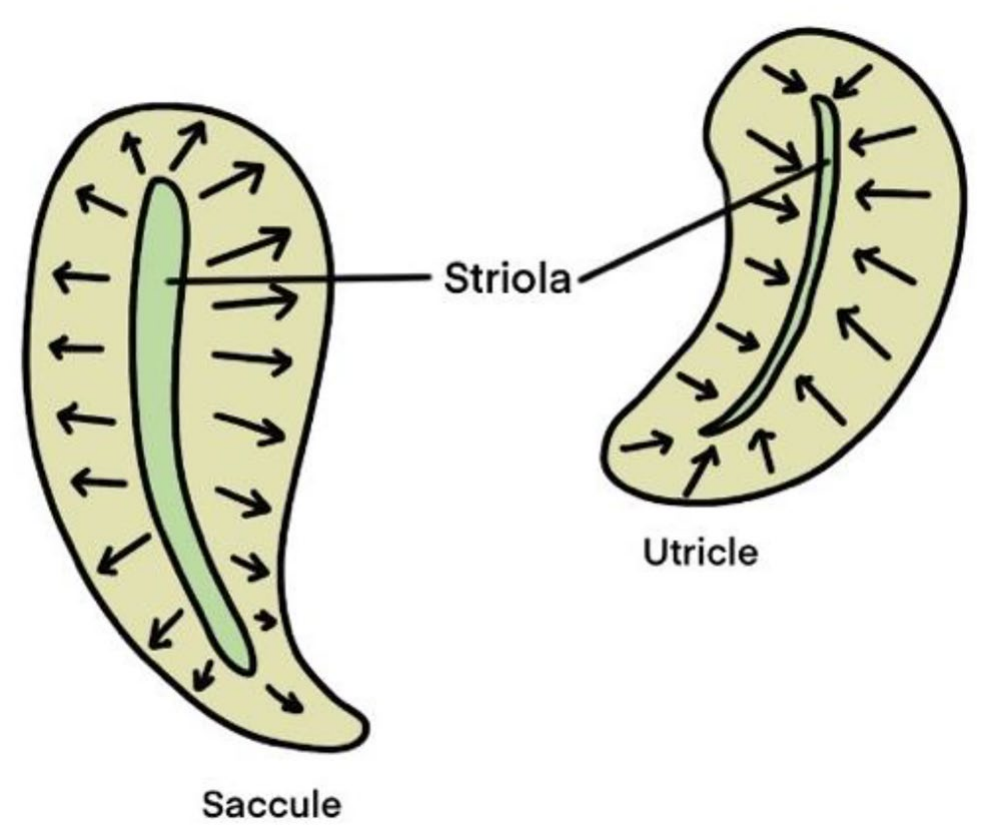
Structure of the striola in saccule and utricle. Cilia of hair cells in the saccule are oriented away from the striola, while cilia of hair cells in the utricle are oriented toward the striola

Vestibular HCs are primary motion sensors in the vestibular sensory epithelium (Kingma and van de Berg 2016). A vestibular HC consists of a cell body and a bundle of cilia on their apical end, which includes a kinocilium, and about 50 stereocilia (Hudspeth and Corey 1977). The kinocilium is the longest cilium, and the closer the stereocilia are to the kinocilia, the longer the stereocilia are. At the top of the cilia is a mechanical connection called elastic tip links, enabling the cilia of one hair cell to move together (Kingma and van de Berg 2016). The striola is a curve for the relative alignment of stereocilia in the macula. The cilia of HCs in the utricle are oriented toward the striola, while cilia in the saccule are oriented away from the striola (Khan and Chang 2013) (Fig. 3). Two types of HCs, Type I hair cells and Type II hair cells, differ in morphology (Ji and Zhai 2018). Type I hair cells have a goblet shape whose bases are surrounded by afferent nerve fibers. Type II hair cells have a columnar shape and the bottom of the cell body connects with the synapses of afferent nerve fibers (Khan and Chang 2013) (Fig. 1).

In response to the stimulation of linear or angular acceleration, the stereociliary bundle of one HC tilts toward the kinocilium, which leads to the activation of the mechanosensitive channel at the tips of the cilia. This can lead to the absorption of K^+^ through top channels of the cilia, depolarizing the membrane of HCs. This depolarization can cause calcium influx by opening voltage-dependent calcium channels at the bases of HCs, stimulating the release of neurotransmitters to increase the firing frequency of nerve fibers (Negishi-Oshino et al. 2019).

## Pathology and mechanisms of vestibular hair cell injury

Generally speaking, human vestibular dysfunction can be attributed to the injury or loss of vestibular HCs. These cells are hypersensitive to certain therapeutic drugs, aging, and genetic factors. Vestibulotoxicity is characterized by the damage or loss of HCs in the utricle, saccule, or semicircular canals.

### Drugs

The main drugs that cause vestibular ototoxicity are anticancer drug cisplatin and aminoglycoside antibiotics.

#### Cisplatin

Cisplatin is a widely used antineoplastic agent (Wu et al. 2017), which has toxic effects on nerves, kidneys, and sensory epithelia in the vestibular and cochlear system (Ding et al. 2018), causing vestibular dysfunction as well as hearing loss. Although cochlear injury of cisplatin has been widely studied, the mechanisms of cisplatin-induced vestibulotoxicity have been relatively less reported (Wu et al. 2017).

It has been reported that cisplatin not only markedly reduced the density of HCs in the utricle, saccule, and ampullae but also gave rise to abnormal morphology and disorganization of vestibular HCs (Tian et al. 2013; Wu et al. 2017; Zhang et al. 2003). Hair cells in the extra-striolar region are more susceptible to cisplatin than those in the striolar area (Cunningham and Brandon 2006). Many studies have suggested that cisplatin-induced vestibular HC death presumably mainly occurs by apoptosis (Kim et al. 2008; Wu et al. 2017; Zhang et al. 2003).

Some studies have noted that cisplatin causes dose-dependent HC loss in the utricle. The number of HCs decreased with the dose of cisplatin increased from 5 to 10 μg/mL (Zhang et al. 2003),0 to 25 μg/mL (Cunningham and Brandon 2006), or from 0 to 50 μg/mL (Lentz et al. 2013). However, Ding et al. reported that the dose–response of vestibular HCs to cisplatin was U-shaped rather than linear. They treated the utricle, saccule, and ampullae of rats with 10–1000 μM cisplatin. When the concentration of cisplatin increased from 10 to 50 μM, the number of vestibular HCs decreased. Conversely, when the dose of cisplatin increased from 100 to 1000 μM, the number of vestibular HCs began to increase. Interestingly, when treated with 1000 μM of cisplatin, almost HCs in cultures were survival, and the maximum HC loss occurred about 50–100 μM (Ding et al. 2018). The author suggested that this might because high levels of cisplatin disrupt the uptake of substances at the top vestibular HCs (Ding et al. 2018). The different dose–response curves of vestibular HCs exposure to cisplatin may be due to the application of a wider concentration range of cisplatin in Ding’s experiment, reflecting the effect of higher doses of cisplatin on vestibular HCs that had not been reflected before.

It has been clarified that the cytotoxicity of cisplatin is associated with the impairment of DNA (Leibbrandt et al. 1995; Ries and Klastersky 1986) and mitochondria (Sugiyama et al. 1989), the accumulation of the reactive oxygen species (ROS) (Matsushima et al. 1998), and the activation of caspase (Kaushal et al. 2001). Previous studies have suggested several possible mechanisms for the vestibulotoxicity of cisplatin as follows:

#### Cisplatin-induced oxidative stress

Monroe et al (2018) reported that after 100 μM cisplatin treatment, the ROS increased dramatically in saccular and utricular tissues of zebrafish. The release of ROS was sharply elevated as cisplatin’s concentrations increased to 500 μM (Monroe et al. 2018). The generation of ROS in the vestibular epithelium caused by cisplatin has been reported to cause vestibular HCs to undergo apoptosis (Tian et al. 2013).

Furthermore, the concentrations of lipid peroxidation (LPO) and nitric oxide (NO) increased significantly after cisplatin treatment (Cheng et al. 2006), which may impair the structure of the cell’s membrane, inhibiting the activation of Na^+^-K^+^ ATPase and Ca^2+^ ATPase of the ampullae of guinea pigs (Cheng et al. 2006). In the vestibular apparatus, Na^+^-K^+^ ATPase and Ca^2+^ ATPase, which are of importance for the transport of K^+^ and Ca^2+^ respectively, can regulate endolymphatic ion distribution and endolymphatic volume. The decrease in Na^+^-K^+^ ATPase’s activity can lead to a reduction in K^+^ concentration in the endolymph (Wangemann 2002). The reduction of Ca^2+^ ATPase’s activity can alter Ca^2+^ concentration in endolymph as well. Ca^2+^ is a prerequisite for the electrical conductivity of HCs and requires exact regulation (Hudspeth 1985). As a result, the physiologic function of vestibular HCs might be affected, as two significant ions, K^+^ and Ca^2+^, get disordered in the endolymph where HCs live and function.

It was reported that iNOS was found in the vestibular end organs after exposure to cisplatin (Watanabe et al. 2000). iNOS can catalyze an insufficient amount of NO, which can react with superoxide and produce peroxynitrites with strong oxidative effects and cytotoxic effects on tissues (Cazevieille et al. 1993; Huie and Padmaja 1993). Therefore, the author concluded that the expression of iNOS and the consecutive unphysiological high NO level might be involved in the vestibular toxicity of cisplatin (Watanabe et al. 2000).

#### Caspase- and caspase-independent cell death pathway

Two signal pathways have been studied to be involved in cisplatin-induced vestibular HC loss. In the vestibular organs exposed to cisplatin, caspase-3 was detected within the cytoplasm of HCs in the utricle, saccule, and ampullae (Wu et al. 2017), and 24 h after cisplatin administration, the expression of caspase-1 and caspase-3 elevated remarkably in both HCs and supporting cells in the utricle of mice (Zhang et al. 2003). The caspase-3 is of significance in the activation of caspase cascade-induced cell apoptosis (Watanabe et al. 2001). Another study showed that it was the p53 that mediated the activation of caspase-3 induced by cisplatin (Zhang et al. 2003).

The caspase-independent apoptotic pathway is also related to the loss of vestibular HCs. The apoptosis-inducing factor (AIF) is a kind of mitochondrial protein, which exists only between the inner membrane and the outer membrane of mitochondria under physiological conditions (Candé et al. 2002a). After exposure to cisplatin for seven days, AIFs could be found in the nucleus of vestibular HCs (Wu et al. 2017), and the rate of AIF nuclear translocation was noticeably increased in vestibular HCs (Wu et al. 2017). After being released from mitochondria, AIFs, capable of binding DNA in the nucleus, can activate the caspase-independent apoptotic pathway when they transfer to the nucleus (Candé et al. 2002a, b).

Some studies have found that some apoptosis-related gene expressions induced by cisplatin increased in HEI-OC1 cell lines (a cochlear hair cell line), including cytochrome c, c-Jun N-terminal kinase (JNK), Bcl-xL, and Bax (Tian et al. 2013). However, it remains unclear whether the activation of these pathways contributes to the loss of vestibular HCs.

## Proinflammatory cytokine expression

Many studies have demonstrated that immune responses can affect vestibular function (Ma et al. 2000). The immune response in the inner ear is crucial for preventing microbial infections, but the increase of cytokines caused by immune responses can damage the inner ear tissue, leading to permanent vestibular dysfunction (Rahman et al. 2001) and hearing loss (Ryan et al. 2002).

Mitogen-activated protein kinases (MAPKs), including JNK and MEK/ERK, can be activated by cisplatin in the vestibular apparatus of mice (Kim et al. 2008). The phosphorylation of JNK and ERK can induce the nuclear translocation and transcriptional activation of NF-kB, one of the critical factors that can regulate the expression of proinflammatory cytokines, thus leading to the mRNA expression and secretion of proinflammatory cytokines. These cytokines include IL-1b, IL-6, and TNF-α and may lead to cisplatin-mediated apoptosis of vestibular HCs (Kim et al. 2008).

### Aminoglycoside

Aminoglycosides (AGs), such as neomycin, gentamicin, and streptomycin, being among the most commonly used antibiotics worldwide, are considered to be ototoxic (Selimoglu 2007). In human beings, gentamicin is thought to be more vestibulotoxic than cochleotoxic (Kim et al. 2013). Compare with cisplatin, aminoglycosides might affect the vestibular system much higher likelihood (Schacht et al. 2012).

The application of AGs can result in a dramatic loss of vestibular HCs (May et al. 2013; Nagato et al. 2018; Yoshida et al. 2015). With the dose of AGs elevated, the rate of vestibular HC loss increased (Lee et al. 2014; Park et al. 2007; Taleb et al. 2008; Yousaf et al. 2018). Apart from the decrease in vestibular HC density, there are also changes at the subcellular level, such as hydropic and vacuolar degeneration of vestibular HCs (Selimoğlu et al. 2003). Regarding HC death caused by ototoxic drugs, cisplatin mainly induces apoptosis, whereas AGs seem to induce both apoptosis and necrosis (Schacht et al. 2012). Some researchers have postulated that apoptosis might occur during the early three days, while vestibular HC death during the latter days may be attributed to necrosis (Hong et al. 2006). Distinct regions of vestibular organs and different HC subtypes have different sensitivity to AGs. The loss of vestibular HCs induced by gentamicin is mainly involved in type I hair cells (Kim et al. 2013; Qian et al. 2021), and HCs in the striolar region are more vulnerable to neomycin than HCs in the extra-striolar region (Cunningham et al. 2002).

The mechanisms of cochlear toxicity of AGs have been illuminated, but vestibular toxicity mechanisms of AGs have been relatively little studied. Existing studies have shown that the ROS is the critical mediator of aminoglycoside-induced vestibular toxicity. AGs exposure can give rise to the formation of ROS in HCs (Hirose et al. 1997), which activates the JNK (Sugahara et al. 2006; Yu et al. 2004). The activation of JNK can lead to the release of cytochrome c from mitochondria into the cytoplasm, and cytochrome c lies upstream of the activation of caspase-9, which can trigger the activation of downstream caspase-3 (Cunningham et al. 2002). Lee et al. pointed out that HC death caused by aminoglycosides may involve a mechanism that the ROS suddenly increase intracellular Ca^2+^ concentration and open up the mitochondrial permeability transition (MPT) pore. Increased intracellular Ca^2+^ levels and the MPT opening are essential elements in the apoptosis caused by aminoglycoside. The resulting cell apoptosis can, in turn, increase intracellular Ca^2+^, thus creating a vicious cycle (Lee et al. 2014).

Other studies have suggested that the increased intracellular Ca^2+^ levels in vestibular HCs induced by AGs might be the consequence of blocking voltage-gated Ca^2+^ channels (Ding et al. 2002). Then, the increased intracellular Ca^2+^ can activate calpains, promoting the degradation of kinases, proteins, and transcription factors (Bartus et al. 1995). Over-activation of calpain will eventually result in vestibular HC death (Kim et al. 2013).

Zhang et al. showed that AIFs were elevated after gentamicin application in vestibular tissue of rats. The amount of AIFs in mitochondria decreased, while the number of AIFs in the cytoplasm increased, suggesting that AIF might be one of the essential factors in apoptosis of vestibular HCs induced by gentamicin (Zhang et al. 2012).

### Aging

Many histopathological studies of human vestibular sensory epithelia have indicated that vestibular HCs degenerate with age (Engström et al. 1977; Lopez et al. 2005; Richter 1980). The HC densities of all five vestibular end organs decreased in subjects with an average age of 84 and 94 years old (Rauch et al. 2001). The declination rate of Type I HCs in the crista ampullaris was higher than that in the macula, whereas there was the same declination rate of type II HCs in all five vestibular end organs(Rauch et al. 2001). However, through an unbiased stereological analysis of 10 subjects with an average age of 82, no age-related HC loss was found in the utricle (Gopen et al. 2003). Differences in the degree of HC degeneration can also be found in different peripheral vestibular apparatus. The loss of HCs in the crista ampullaris was greater than that in the macula (Anniko 1983), and there was a more significant loss of HC in the saccule than that in the utricle (Rosenhall 1973). There are also some underlying age-related changes in vestibular HCs, including the loss and disorder of cilia, the accumulation of lipofuscin pigments, the appearance of intracellular vesicles, the disruption of the cuticular plate (Anniko 1983; Richter 1980), as well as some mitochondrial changes, such as the abnormal morphology and the increased number of mitochondria, and the disintegration of mitochondrial crista (Baloh et al. 1997).

Age-related hearing loss is now recognized as a disorder caused by a combination of many internal genetic factors and external environmental factors (Ohlemiller 2009; Yamasoba et al. 2013). By comparison, little known is about mechanisms behind the changes of HCs in vestibular sensory epithelia. However, based on multiple relevant research, some possible reasons have been suggested. With aging, the number of mtDNA copies has been reported to decrease by 50–70%, and gene expression associated with oxidative phosphorylation (OXPHOS) is also reduced. Based on these facts above, one reason may be that vestibular HCs that are considered to be heavily dependent on OXPHOS for ATP (Spinelli et al. 2012) may become more sensitive to ototoxic factors due to this potential energy crisis (Bigland et al. 2018). Other researchers have suggested that the accumulation of calcium and ROS might be of significance in the age-related injury of vestibular HCs (Brosel et al. 2016). This hypothesis was supported by the following facts: (1) Aging mice showed a decrease in several proteins, including TRPV5, TRPV6, and Klotho (Tanaka et al. 2012). These proteins are significant to Ca^2+^ metabolism in vestibular and cochlear tissues, and the normal function of HCs depends on low concentrations of calcium in the endolymph. Besides, Klotho can attenuate oxidative stress (Yamoah et al. 1998); (2) With age, some oxidative stress markers such as nitrotyrosine and hydroxynonenal in the inner ear increased. Meanwhile, SOD2, one of the important antioxidant enzymes, decreased (Yamoah et al. 1998). This oxidative stress may result in mitochondrial dysfunction and result from the continuously increased deletions of mitochondrial DNA, leading to a vicious cycle (Crawley and Keithley 2011; Markaryan et al. 2008).

### Genetic factors

Genetic factors can contribute to vestibular HC loss. The MyoXVa: whirlin: Eps8 complex at the tips of stereocilia is critical for the elongation of stereocilia (Manor et al. 2011). The role of whirlin is to help the MyoXVa: Eps8 complex assemble properly and increase its stability (Manor et al. 2011). Eps8 directly regulates the role of whirlin in stereocilia (Manor et al. 2011). Myosin VI functions as a connection between stereocilia and the cuticular plate (Hasson et al. 1997). As a result, the mutation of genes related to stereociliary function can result in the damage of vestibular HCs. The vestibular HCs of Eps8-KO mice showed significantly shorter stereocilia (Tavazzani et al. 2016). The whirler mouse, a model of human Usher syndrome, which cannot produce functional whirlin proteins encoded by WHRN in the inner ear (Mathur et al. 2015b), manifested abnormally shortened stereocilia of both cochlear and vestibular HCs (Holme et al. 2002; Mburu et al. 2003). Furthermore, both Dfnb31neo/neo mice, which has a deletion between Dfnb31(WHRN) exons 6 and 9, and Dfnb31wi/wi mice, which has a mutation in Dfnb31 exon 1, were characterized by shortened stereocilia of the macula (Mathur et al. 2015a).Tur/Tur mutant mice, which has a point mutation in exon 8 of the Myo6 gene, showed a significant reduction of HCs in the three cristae. Their stereociliary bundles seemed to be elongated, distorted, and disordered in the macula (Wong et al. 2016). It has been observed that mice with a deletion of exon 5 in the Myo6 gene exhibited elongated and fused stereocilia at P2, and even complete absence of stereocilia at eight weeks (Williams et al. 2013).

Apart from this, some studies also found additional genes, without which vestibular HCs cannot maintain in a normal state. Ablation of the function of prosaposin (a precursor of four glycoprotein activators for lysosomal hydrolases) led to the overgrowth of afferent and efferent neurons below vestibular HCs and these excess neurons can destroy HCs and supporting cells in the vestibular end organs of mice (Akil and Lustig 2012). After deletion of Brg1, an ATPase subunit of a chromatin-remodeling complex, vestibular HCs of mice exhibited fused and elongated stereocilia bundles, and most HCs with fused stereociliary bundles did not have a cuticular plate (Akil and Lustig 2012). A mutation in the SLC26A4 gene can cause extensive HC loss and degeneration in the saccule, utricle, and crista of mice (Lu et al. 2011). Mice with a defect of Brn-3c showed a substantial loss of HCs in cochlear and vestibular epithelia during the third trimester and early postnatal period (Xiang et al. 1997).

## Protection strategies of vestibular hair cells

Currently, practical strategies for preventing the loss or damage of vestibular HCs mainly focus on pharmacotherapy and gene therapy.

### Pharmacotherapies

#### Heat shock protein

Various kinds of cellular and environmental stress can induce the expression of heat shock proteins (HSPs). The induction of HSPs is a ubiquitous and highly conserved stress response that can inhibit apoptosis in numerous systems (Martindale and Holbrook 2002). Some studies have argued that heat shock-induced HSPs inhibit aminoglycoside-induced (Taleb et al. 2008) and cisplatin-induced (Baker et al. 2015) HC death in mouse utricle. Both HSP 70 and its major heat shock transcription factor, HSF1, are necessary for performing this protective effect (Baker et al. 2015), and some studies have observed that HSP70 is mainly secreted by supporting cells, not by HCs in heat shock responses in the inner ear (May et al. 2013). Although constitutive expression of HSP70 brings about a robust protective effect against HC loss from aminoglycoside treatment (Taleb et al. 2008), it provides only modest protection against HC death induced by cisplatin (Baker et al. 2015). Overexpression of HSP70 inhibits vestibular HC death from ototoxicity as well (May et al. 2013). Furthermore, some factors, which can induce HSP70 in the vestibular end organs, such as geranylgeranylacetone (GGA) (Nagato et al. 2018; Takumida and Anniko 2005) and pifithin-µ (Ryals et al. 2018), also protect vestibular HCs from aminoglycoside-induced death. HSP32 is another heat shock protein that may prevent vestibular toxicity induced by cisplatin, and its protective effect is mediated by resident macrophages (Baker et al. 2015). Celastrol, a traditional Chinese medicine, can induce HSP32 through activating the Nrf2-transcription factor (Francis et al. 2011), and HSP32 can inhibit pro-apoptotic JNK activation and HCs death (Francis et al. 2011). In summary, these studies above underscored the beneficial effects of HSP70, HSP32, and their inducer against the ototoxic drug-induced vestibular HC loss. However, mechanisms by which HSP protects against vestibular HC loss need further investigation in the future.

#### Antioxidant treatment

For protecting vestibular HCs from damage, antioxidants have potential therapeutic effects. Metformin, which can greatly reduce the release of ROS induced by gentamicin, inhibited gentamicin-induced apoptosis of HCs in rat utricle (Lee et al. 2014). Renexin (RXN), a combination of Ginkgo biloba extract (GbE) and Cilostazol (CS), noticeably attenuated cisplatin-mediated ROS production in HEI-OC1 cells (Tian et al. 2013). When combined with cisplatin, RXN kept stereocilia in the rat utricle and saccule preserved and well-shaped (Lee et al. 2014). Owing to a deletion mutation in the γ-glutamyl transferase-1 gene, dwarf gray mice cannot encode an enzyme essential for glutathione resynthesis, giving rise to the loss of vestibular HCs and large vacuoles in vestibular HCs of dwarf gray mice (Ding et al. 2016). Hydrogen gas can effectively protect vestibular HCs against morphological and functional damage caused by antimycin A by reducing ROS (Taura et al. 2010). Collectively, these studies indicated that antioxidants are a promising approach for rescuing vestibular HC loss caused by ototoxic drugs.

Other factors have been shown to reduce cisplatin-induced ROS in vestibular organs, including D-methionine (Cheng et al. 2006), which can reduce cisplatin-induced LPO and NO in the vestibular HCs of guinea pigs, and EF-24(bis[(2-fluorophenyl)methylene]-4-piperidinone) (Monroe et al. 2018) which can decrease ROS release by cisplatin treatment in the maculae of zebrafish. However, further investigation is required to understand the protective effects of D-methionine and EF-24 on vestibular HCs. Both the L-NG-nitroarginine methyl ester (L-NAME) (Takumida and Anniko 2002; Takumida et al. 2003), a non-special NOS inhibitor, and D-methionine (Takumida et al. 2003), a radical scavenger, both increased the survival rate of vestibular HCs after the treatment of gentamicin. Some studies (Hoshino et al. 2011; Kim et al. 2015) have noted that Nrf2, an antioxidant factor, can prevent cochlear HC loss from ototoxic drugs and age-related damage. Kumiko et al. demonstrated that Nrf2-IR is also present in HCs and supporting cells of five vestibular end organs (Hosokawa et al. 2018). However, the protective effect of Nrf2 in vestibular HCs needs further explorations.

#### Inhibition of programmed cell death

The use of diverse antiapoptotic agents to manipulate intrinsic cell death is also a promising intervention for protecting vestibular HCs. Several scientific research has reported that the MAPK/JNK pathway plays an essential role in the death of cochlear HCs and vestibular HCs caused by aminoglycosides (Sugahara et al. 2006). In animal models, blocking this pathway with the JNK inhibitor CEP11004 considerably protected utricular HCs treated with intermediate doses of neomycin (Sugahara et al. 2006), but it did not show any protective effect against high doses of neomycin-induced vestibular HC death (Sugahara et al. 2006). A study disclosed that allicin inhibited caspase-dependent and caspase-independent apoptosis pathways and protected cisplatin-induced vestibular HC loss (Wu et al. 2017). Inhibition of caspase activation by the administration of the pan-caspase inhibitor zVAD promoted the survival of vestibular HCs after aminoglycoside treatment in chicks (Matsui et al. 2003).

#### Growth factors

Insulin-like growth factor-1 (IGF-1) is considered to be one of the essential regulators for the growth and development of the inner ear (Varela-Nieto et al. 2004), and many studies have shown its vestibular protective effect for preventing vestibular HC loss by aminoglycoside exposure (Angunsri et al. 2011; Park et al. 2007; Yoshida et al. 2015). Extensive loss of HCs in the utricle caused by neomycin was rescued by IGF-1 treatment (Angunsri et al. 2011). Parallel to IGF-I, IGFBP(insulin-like growth factor binding protein)-4 and IGFBP-5 are significantly protective (Park et al. 2007). IGF-I, IGFBP-4, IGFBP -5, and the combination of IGF-I and IGFBP-5 increased HC survival in mouse utricle after neomycin application (Park et al. 2007). However, the combination of IGFBP-4 and IGF-I is less effective than applying each one alone (Park et al. 2007). The SSSR, which is the minimum peptide of IGF-1, and the combination of SSSR and FGLM-NH2 which is the smallest peptide of SP, have also been reported to be protective against neomycin-induced vestibulotoxicity (Yoshida et al. 2015). However, the mechanisms underlying those protective effects remain unclear, but there are some hypotheses: (1) Unlike HCs of cochlear epithelia, vestibular HCs of mammals can regenerate to some extend (Taura et al. 2006), and IGF-1 may facilitate this regeneration (Kopke et al. 2001) to protect vestibular HCs (Park et al. 2007); (2) Aminoglycoside-induced HC loss is associated with activation of JNK (Zheng et al. 1997) and IGF-1 in the inner ear can modulate JNK activation, thereby inhibiting aminoglycosides-induced HC death. However, additional experiments are required to confirm these hypotheses. Treatment with brain-derived neurotrophic factor (BDNF) also has protective effects against vestibular HC degeneration by gentamicin (Takumida and Anniko 2002; Takumida et al. 2003).

#### Other protective agents and molecules

Excepted from what has been stated above, there are other treatments for vestibular HC loss. Leupeptin, a calpain inhibitor, noticeably inhibited gentamicin-induced density reduction of HCs in the utricle and crista ampullaris of mice induced by gentamicin (Ding et al. 2002). Tacrine has also been elucidated to protect the utricular HCs of mice against neomycin (Ou et al. 2009). When pretreated with tacrine, HCs from both striolar and extrastriola regions were retained after applying neomycin. P2X2 purinergic receptors in vestibular transitional cells (Lee et al. 2001) have been reported to carry cation currents, thereby protecting utricular HCs during increased stimulation intensity (Jeong et al. 2020). Korean red ginseng has been proven to protect against age-related HC loss in five periphery vestibular organs (Tian et al. 2014).

#### The combination of different pharmacotherapies

It has been indicated in some studies that the protective effect of a drug combination is better than using one drug alone. Treatment with combinations of L-NAME + BDNF (Takumida and Anniko 2002; Takumida et al. 2003), L-NAME + leupeptin (Takumida et al. 2003), and D-methionine + BDNF (Takumida et al. 2003) can save more vestibular HC after exposure to gentamicin than using any one of them alone. So researchers suggested that the combinations of ROS scavengers with neurotrophic factors or ROS scavengers with calpain inhibitors may yield greater therapeutic effects for vestibular diseases (Takumida et al. 2003).

#### Gene therapy

Gene therapy refers to the insertion of foreign genes into the appropriate receptor cells of patients through gene transfer technology, so that the product produced by extraneous genes can achieve a desired therapeutic outcome. It provides an opportunity to repair mutated genes before birth, thus antagonizing the morphological or functional insufficiency of vestibular HCs caused by some congenital gene-related diseases. At present, in vestibular HC protection, gene therapy has been relatively widely used in the treatment of Usher syndrome. Delivering gene products to vestibular HCs via adeno-associated virus (AAV), the distorted stereociliary bundles due to the Whirlin mutation in an Usher2d mouse model was successfully restored, and the survival rate of HCs increased in both the cochlea and utricle (Chien et al. 2016; Isgrig et al. 2017). Lentz et al. saved vestibular and cochlear HCs of Usher1c mouse by correcting pre-mRNA splicing defects in Usher1c mouse model with antisense oligonucleotides (Lentz et al. 2013). Gene therapy mediated by AV2/Anc80L65 restored mechanical transduction of vestibular HCs in another Ush1c mutant mouse model (Pan et al. 2017). The stereociliary length of whirler mice was successfully restored after injecting AAV8-whirlin into posterior semicircular canals (Isgrig et al. 2017).

## Conclusions

For the past few years, a majority of studies on vestibular HCs have mainly focused on the development of multiple therapeutic strategies to protect vestibular HCs from destruction and reverse vestibular dysfunction. Numerous vestibular protective measurements, consisting of heat shock proteins and their stimulants, antioxidants, anti-inflammatory agents, manipulation of intrinsic pathways of cell death, growth factors, and gene therapy, are likely to provide an impetus for the comprehensive development of vestibular HC protection in the future. Furthermore, understanding the mechanisms of vestibular HC toxicity caused by distinct factors can also help develop effective treatments aiming at these mechanisms.

In addition to the protective agents targeting the mechanisms of vestibular HC damage mentioned in this review, such as antiapoptotic and antioxidant drugs, to our knowledge, some mechanisms that cause vestibular HC damage have not been reported with targeted drugs. In the future, attempts can be made in these mechanisms, such as whether antagonizing the increase of inflammatory cytokines induced by cisplatin can save cisplatin-induced vestibular HC loss; Whether overexpression of mtDNA can prevent energy deficiency caused by the loss of mtDNA due to vestibular HC aging.

Additionally, compared with vestibular HCs, the factors leading to the loss of cochlear HCs and their mechanisms, as well as the corresponding therapeutic strategies, have been relatively clarified. With histological correlations between cochlear and vestibular HCs, we can verify whether these factors, mechanisms, and treatments have proven to be effective in cochlear HCs by multiple existing studies but have not yet been applied to vestibular HCs, can be used to vestibular HCs in the future. For example, whether noise and viral infection that damage cochlear HCs will lead to vestibular HC loss; Whether the PI3K-Akt pathway causing the apoptosis of cochlear HCs is involved in the apoptosis of vestibular HCs, and whether the mutation in Pou4f3 gene and mtDNA that increases the susceptibility to ototoxic drugs of cochlear HCs can increase the sensitivity to ototoxic drugs of vestibular HC; Whether some antioxidants such as N-acetylcysteine, MitoQ, Nrf2, and antiapoptotic agents, such as KNUA002, DUSP1, which have been elucidated to protect cochlear HCs, can perform similar protective effects on vestibular HCs. Moreover, whether some strategies used to preserve cochlear HCs, such as chemical modification of ototoxic drugs, application of non-coding RNA, inhibition of the uptake of toxic factors, regulation of autophagy of HCs, and manipulation of epigenetics such as methylation and acetylation, can effectively protect vestibular HCs.

Furthermore, with the progress of emerging technologies, novel methods can be used for further investigation. For example, scRNA-seq can be used to identify more genes regulating vestibular HC damage; Epigenomics and proteomics can be used to ascertain the mechanisms of various injury factors leading to vestibular HC loss; The library of integrated cellular signatures and bioactive compounds can be used to recognize new otoprotective agents; CRISPR/Cas9 can be used to treat gene-related vestibular HC degeneration.
