# Correction to: Progress in protecting vestibular hair cells

**DOI:** 10.1007/s00204-021-03117-w

**Published:** 2021-07-09

**Authors:** Luoying Jiang, Zhiwei Zheng, Yingzi He

**Affiliations:** 1 ENT Institute and Department of Otorhinolaryngology, Eye and ENT Hospital, State Key Laboratory of Medical Neurobiology and MOE Frontiers Center for Brain Science, Fudan University, Shanghai, 200031 China; 2 NHC Key Laboratory of Hearing Medicine, Fudan University, Shanghai, 200031 China

## Correction to: Archives of Toxicology 10.1007/s00204-021-03067-3

The authors found mistakes in 2 figures of their publication "Progress in protecting vestibular hair cells ", published online 13 May 2021. They have redrawn Fig. 2, correcting the wrong notes, and they have optimized the drawing of nerve fibers in both Figs. 1 and 2, showing afferent and efferent nerve fibers at the same time.

1. In the right notes of Fig. 2, the “Type I hair cell” and “Type II hair cell” are switched.
2. There add efferent nerve fibers at the bottom of hair cells in both Figs. 1 and 2.

**Fig. 1:**
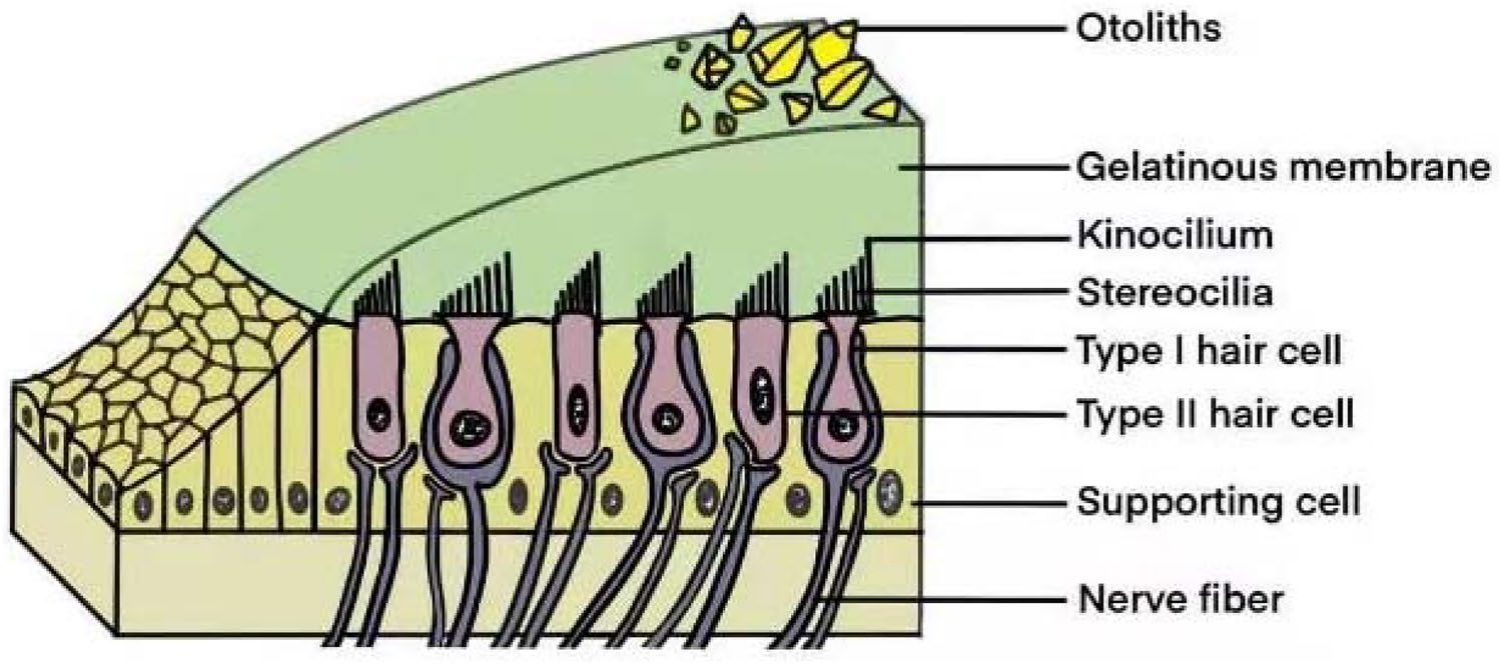
Structure of the macula. The macula is composed of the otolithic membrane and macular epithelium. The otolithic membrane consists of a gelatinous membrane and some otoliths. The macular epithelium comprises sensory hair cells and supporting cells

**Fig. 2:**
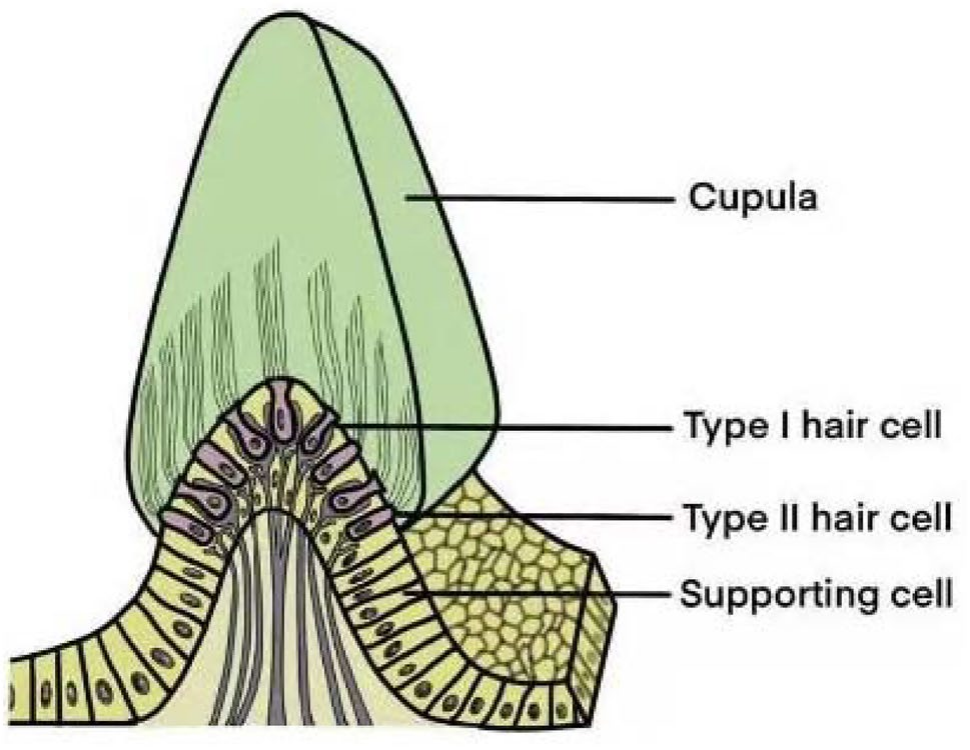
Structure of the crista ampullaris. The histological morphology of the crista ampullaris is similar to the macula, while its gelatinous membrane called the cupula has no otoliths and is thicker than that of the macula

